# Selective head–neck cooling after concussion shortens return-to-play in ice hockey players

**DOI:** 10.2217/cnc-2021-0002

**Published:** 2021-04-15

**Authors:** Anna Gard, Yelverton Tegner, Mohammad Fazel Bakhsheshi, Niklas Marklund

**Affiliations:** 1 Lund University, Skåne University Hospital, Department of Clinical Sciences Lund, Neurosurgery, Lund, Sweden; 2 Department of Health Sciences, Luleå University of Technology, Luleå, Sweden; 3 Lund University, Family Medicine & Community Medicine, Lund, Sweden; 4 BrainCool AB, Medicon Village, Lund, Sweden

**Keywords:** brain temperature, concussion, hypothermia, ice hockey, return to play, selective head–neck cooling, sports-related concussion, traumatic brain injury

## Abstract

We aimed to investigate whether selective head–neck cooling could shorten recovery after sports-related concussions (SRCs). In a nonrandomized study of 15 Swedish professional ice hockey teams, 29 concussed players received immediate head and neck cooling for ≥30 min (initiated at 12.3 ± 9.2 min post-SRC by a portable cooling system), and 52 SRC controls received standard management. Players receiving head–neck cooling had shorter time to return-to-play than controls (7 vs 12.5 days, p < 0.0001), and 7% in the intervention group versus 25% in the control group were out of play for ≥3 weeks (p = 0.07). Immediate selective head–neck cooling is a promising option in the acute management of SRC that should be addressed in larger cohorts.

Sports-related concussions (SRCs) are common in contact sports such as football, soccer, rugby and ice hockey [1,2]. The incidence has increased in many sports, including ice hockey [3,4]. The majority of SRCs lead to relatively short-lived symptoms, and most athletes return to play (RTP) within a couple of weeks [5,6]. However, some 15–20% of concussed athletes have a prolonged recovery, up to 30% in ice hockey [7,8,9], and a few percentage of athletes develop persisting postconcussion symptoms [9,10]. Additionally, repeated SRCs have been associated with depression, premature onset of Alzheimer disease and neurodegenerative disorder [4,10,11].

Although the initial symptoms after an SRC, defined as a mild traumatic brain injury (mTBI), are caused by a functional disturbance, refined neuroimaging studies also reveal structural injuries commonly persisting beyond symptom resolution [6,12]. Regardless, an energy crisis may occur at time of SRC due to energy metabolic alterations caused by reduced cerebral blood flow, increased glucose consumption and a disrupted blood–brain barrier [13,14]. After a TBI, mitochondrial energy metabolism is disturbed, potentially acting as a strategy for neuroprotection via a decrease in energy consumption [15]. These energy metabolic perturbations may result in increased vulnerability to the brain during the initial postinjury time period. Thus, accurate diagnosis and management at the time of SRC, including removal of the athlete from play, might reduce the risks of repeated injury and prolonged recovery [16].

Increased brain temperature is a well-established secondary injury insult in TBI [17,18]. For every degree raise in temperature, the brain's demand for oxygen and glucose increases by 6–10% [19]. Furthermore, elevated body temperature is associated with worse outcome in acute brain injuries such as stroke and intracerebral hemorrhage [20]. In strenuous sports such as ice hockey, the core body temperature is markedly elevated [21], and exercise increases cerebral metabolic heat production [22]. An SRC occurring at time of elevated core (and probably brain) temperature may thus lead to worse outcome [23]. Supporting the concept of increased brain vulnerability, a larger cortical lesion and exacerbated neuronal loss following mTBI in rats with pre- and post-injury moderate (39°C) hyperthermia compared with normothermic animals was found [23]. Furthermore, cognition was impaired in hyperthermic rats after mTBI [24].

For decades, there has been a clinical interest in the use of hypothermia for the treatment of various CNS disorders. In animal TBI models, hypothermia consistently improves histological outcome [25], and this archetypical neuroprotectant has also been applied in several clinical TBI trials aiming at 32.0–36.5°C. In severe TBI, it effectively reduces intracranial pressure (ICP). However, on the basis of a meta-analysis of more than 3000 patients as well as two recent clinical trials [26,27,28], clinical outcome was not improved, and whole body cooling is not standard of care in severe TBI. Selective brain cooling was attempted in a small, single-center trial in China, showing reduced ICP and beneficial outcomes up to 2 years postinjury [29]. In repetitive mTBI as a model of SRC, mild hypothermia provided axonal and microvascular protection in rats [30], and when applied within 15 min post-mTBI in hyperthermic rats, cognitive function was improved [24]. These studies provided the rationale for applying selective head–neck cooling for the acute treatment of SRC.

Although early rest and a graduated RTP strategy is accepted following SRC, specific initial treatment options are scarce [6]. A rise in brain temperature increases brain metabolism and exacerbates neuronal injury in a variety of acute CNS disorders including TBI, and hypothermia has been evaluated in numerous clinical studies [19,31]. When aiming at a brain temperature of 32.0–36.5°C using various cooling methods, intracranial pressure was decreased in severe TBI, although without beneficial effects on clinical outcome [26]. Instead, aggressive reduction of elevated core temperature aiming at normothermia is generally accepted.

Ice hockey players have demonstrated elevated body temperature during play [21], and body temperature approaching 40°C has been observed in contact sports [32]. Brain and body temperatures are strongly correlated, where brain temperature is slightly higher [33,34]. When an athlete sustains an SRC, defined as an mTBI, elevated brain temperature may predispose for a more extensive brain injury [21,23,33]. Because systemic cooling aimed at inducing hypothermia is not practical for mTBI, selective head–neck cooling may be an alternate strategy after SRC.

In recent decades, SRC has become an growing problem in Swedish ice hockey [3], and improved treatments are urgently needed. In this study, our aim was to investigate whether early selective head–neck cooling treatment improves outcome in Swedish professional ice hockey players.

## Material & methods

### Ethics

All research described herein was approved by the regional ethics committee in Lund, Sweden (decision nos. Dnr 2015/658, Dnr 2016/804, Dnr 2016/941, Dnr 2017/125 and Dnr 2018/1019). Written informed consent was obtained from study participants.

The study is registered at ClinicalTrials.gov (protocol record *NCT04701125*).

### Study participants & study design

**Figure 1.**
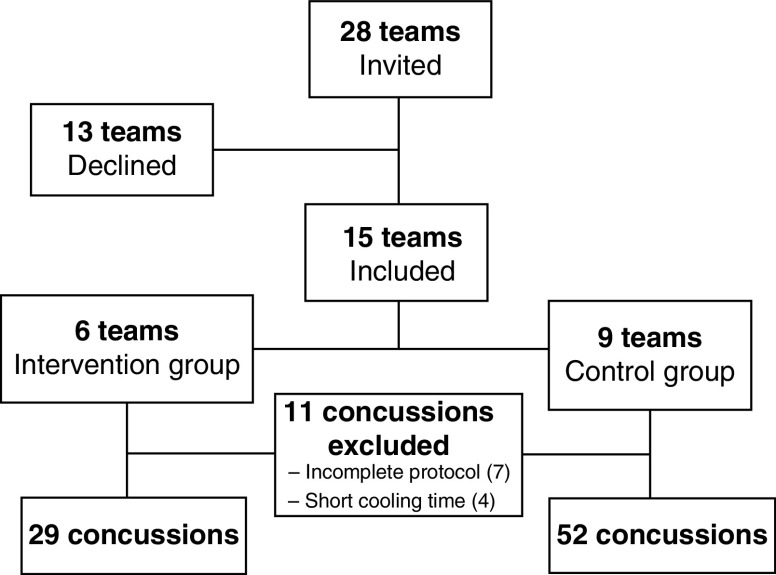
Consort flow diagram.

Twenty-eight teams from the elite ice hockey leagues for males in Sweden (first league, the Swedish Hockey League; second league, the HockeyAllsvenskan) were invited to participate in the study. The Swedish Hockey League is the highest ranked league, whereas all players in the HokeyAllsvenskan are professional or semiprofessional. Fifteen teams agreed to participate. The number of players on each team varies from around 24 to 30. Participating teams were given the option to be in the study group (receiving selective head–neck cooling after SRC) or the control group (standard SRC management) (Figure 1). The medical teams of each elite ice hockey team had undergone thorough training on early concussion management, graduated RTP protocols and the rehabilitation of a concussed player. A baseline questionnaire on previous SRCs was conducted in each team.

Any player with suspected or diagnosed concussion was immediately removed from practice or game participation for further evaluation. Concussion diagnosis was established using the concussion in sport consensus statement from Zurich, 2012 [35]. According to this definition, a concussion was defined as: transient neurological symptoms (e.g., loss of consciousness, memory loss, blurred vision, dizziness, balance problems and confusion) caused by an external force/trauma to the head, either direct or indirect. Evaluation was made by the person medically responsible of each team. At time of SRC, any presence of ‘red flags’ mandated the player to be taken to the hospital. Red flags include unconsciousness, seizures, focal neurological deficits, rapidly worsening headache, numbness/tingling or weakness in hands, neck pain, slurred speech and repeated vomiting.

As soon as the diagnosis of SRC was established, the player was included in the study and players in the intervention teams were treated by selective head–neck cooling using the PolarCap System (see the next section for more information on the cooling system) for a minimum of 30 min. A delay of up to 3 h from the time of SRC to initiation of the cooling procedure was acceptable; this time range was decided before the study onset. Teams in the first and second leagues were invited to participate in the study and were all offered the possibility of using the cooling device. Athletes playing for a team that chose to participate in the study and who also agreed to participate after an SRC were included in the study. Athletes who did not adhere to the cooling procedure, had red flags or denied participation were excluded. The primary endpoint of the study was time (in days) from concussion until returning to full practice or game, that is, return to play.

### Cooling procedure

**Figure 2.**
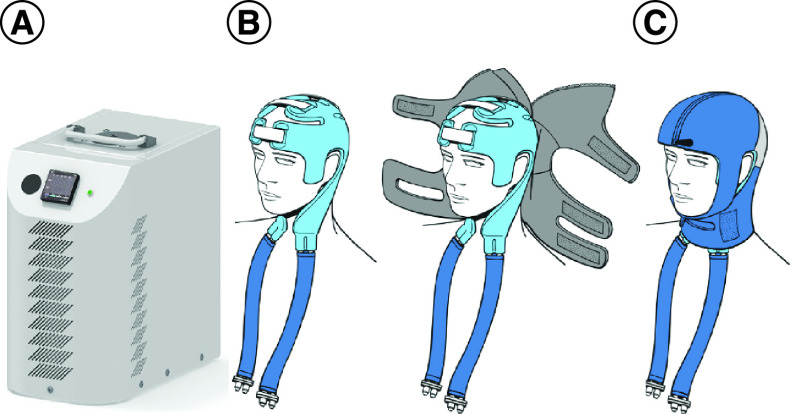
Selective head-neck cooling system. **(A)** The PolarCap^®^ System is a high-powered portable cooling system. **(B)** The PolarCap^®^ Coolant flows through a silicone-based head cap. **(C)** An insulating neoprene cover is put on top of the cap to isolate the cold.

The PolarCap System consists of a high-powered portable cooling system (Figure 2A), designed to reduce brain temperature by controlled cooling of the scalp and neck with a circulating coolant (PolarCap Coolant, PolarCool AB, Lund, Sweden). The coolant is continuously controlled to be maintained at 0°C to avoid reaching too low temperatures and maintain the desired temperature. The PolarCap Coolant flows through a silicone-based head cap (Figure 2B). An insulating neoprene cover is put on top of the cap to isolate the cold (Figure 2C). The players were allowed to relax (sitting or supine) as long as the cooling head cap and neoprene cover remained on their head. Time from injury to treatment start and cooling duration were noted in the study protocol. Players were asked to use the system for a least 30 min. The medical staff for each team was trained in how to apply the device, then initiated and supervised the cooling at the time of SRC. Any side effects of treatment were noted in the study protocol.

### Post-SRC management

All players who sustained a SRC were put on a rehabilitation program including the standardized graduated RTP protocol. The graduated RTP protocol began with 24–48 h of ‘brain rest’ with minimal physical and mental activity, followed by a gradual, stepwise increase in activity. The time between each step was at least 24 h. This program has been used for several years in Swedish ice hockey and was familiar to the teams [36,37]. Time until the medical teams judged the players ready for game play, as evaluated by player symptoms, was recorded. Data were reported by team physicians on a study protocol.

### Effects of head–neck cooling on measured temperatures

To determine whether temperatures were affected by selective head–neck cooling using the PolarCap System, three healthy volunteers were evaluated using measurements of body and subdermal forehead temperature before, during and after exercise. Body temperatures were measured by gastrointestinal (GI) capsule systems. The e-Celsius system consisted of a GI capsule and a receiver, referred to as the monitor (E-Celsius Performance, BodyCap Medical, Hérouville-Saint-Clair, France). Data were stored and transmitted every 30 s to the monitor. The capsule was ingested at least 2 h before exercise. Zero-heat-flux (ZHF) thermometry is accepted as an accurate measurement of subdermal temperature [38,39,40], and such sensor patches were placed in the forehead a few centimeters from the cooling system.

Each volunteer did two trials, one with and one without the cooling system. After exercise, in the first trial the volunteer rested, and in the second trial received selective head–neck cooling using the PolarCap System. Before starting the cooling process, the cooling unit that controlled the temperature of cooling bath was set to 0–3°C for 30–45 min; settings were kept unchanged during the experiment. Temperatures were recorded manually at 1-min intervals.

### Statistical analysis

Data were entered into a Microsoft Excel (Microsoft Corporation, WA, USA) spreadsheet and analyzed with Minitab 19 (Minitab Inc., PA, USA). A two-sample Wilcoxon rank–sum test was used to assess for difference in the median RTP values for the control and intervention group. For calculating the proportion of players absent for 3 weeks or more, Fisher's exact test was used. A two-sample t-test was used to assess difference in concussion history.

## Results

**Figure 3.**
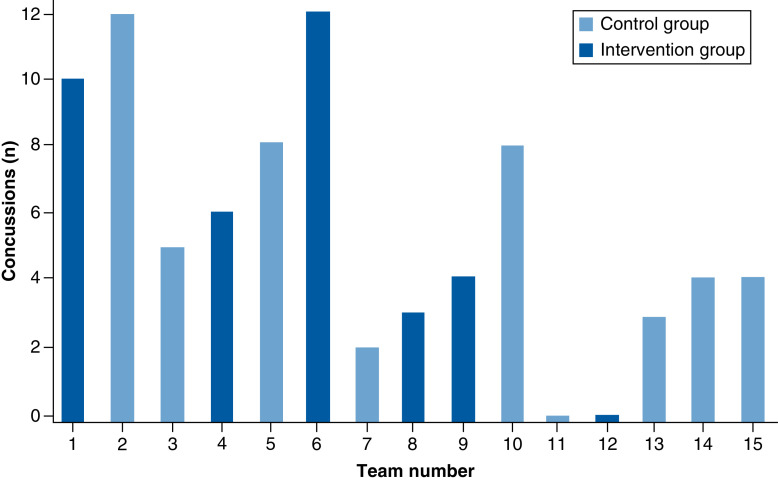
Concussions per team.

Fifteen ice hockey teams, 11 in the first league (SHL, of which four were in the intervention group), two in the second league (HockeyAllsvenskan, both of which were in the intervention group) and two that played in both the first and second leagues during the study period (both of which were in the control group) were enrolled in the study. The reasons for not using the head–neck cooling system included logistic reasons, hesitation regarding the feasibility of implementing the intervention and skepticism regarding its efficacy. Over the seasons 2016–2017, 2017–2018, and 2018–2019, 92 players were diagnosed with a concussion. Due to incomplete protocols (n = 7) or that the cooling time was shorter than the prespecified duration of 30 min (n = 4), 11 protocols were excluded. Thus, 81 SRCs were available for analysis (Table 1). No included player had a red flag symptom mandating immediate transfer to hospital. Number of concussions and the level of play (first second leagues) of the concussed player is shown in Figure 3. The number of reported concussions varied substantially, where two teams did not report any concussion in any season during the study (Figure 3).

**Table 1.** Concussions per season.

	2016–2017	2017–2018	2018–2019	Total
Intervention	7	14	8	29
Control	8	7	37	52
Total	15	21	45	81

**Figure 4.**
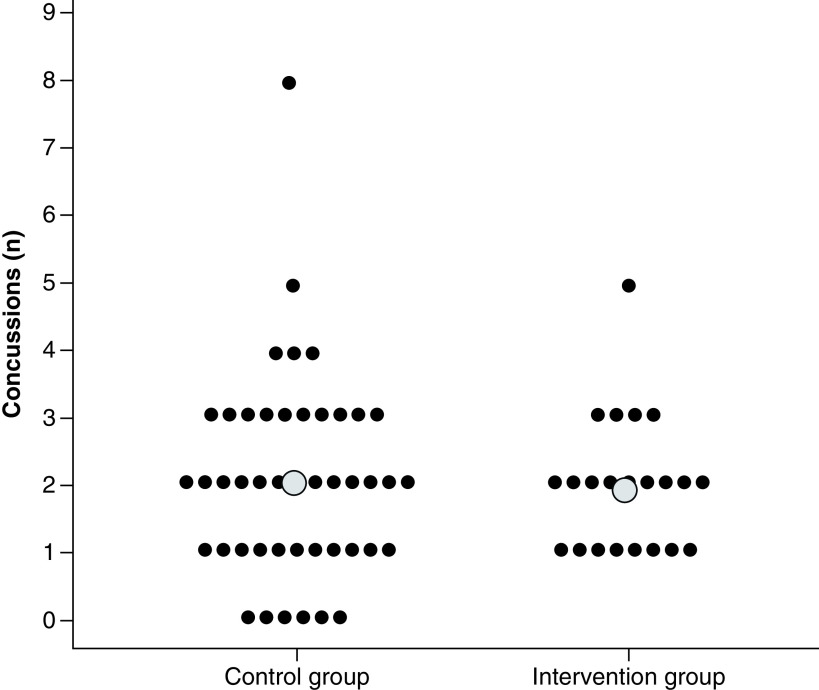
Previous concussions. Number of previous concussion (number/player) in the control and intervention (head–neck cooling) groups. Mean value is indicated by a larger gray dot. There were no differences between the groups.

The age of the players was similar in the intervention group (24.8 ± 5.6 years old) and the control group (25.6 ± 4.6 years old). The number of previous concussions was equally distributed between the intervention and control groups (a mean of 1.8 ± 1.3 vs 2.0 ± 1.5 previous concussions per included player, respectively; Figure 4).

The time from SRC to initiation of cooling was a mean 12.3 ± 9.2 min, range: 3–35 min. The cooling time was 46 ± 12 min, range: 30–70 min.

**Figure 5.**
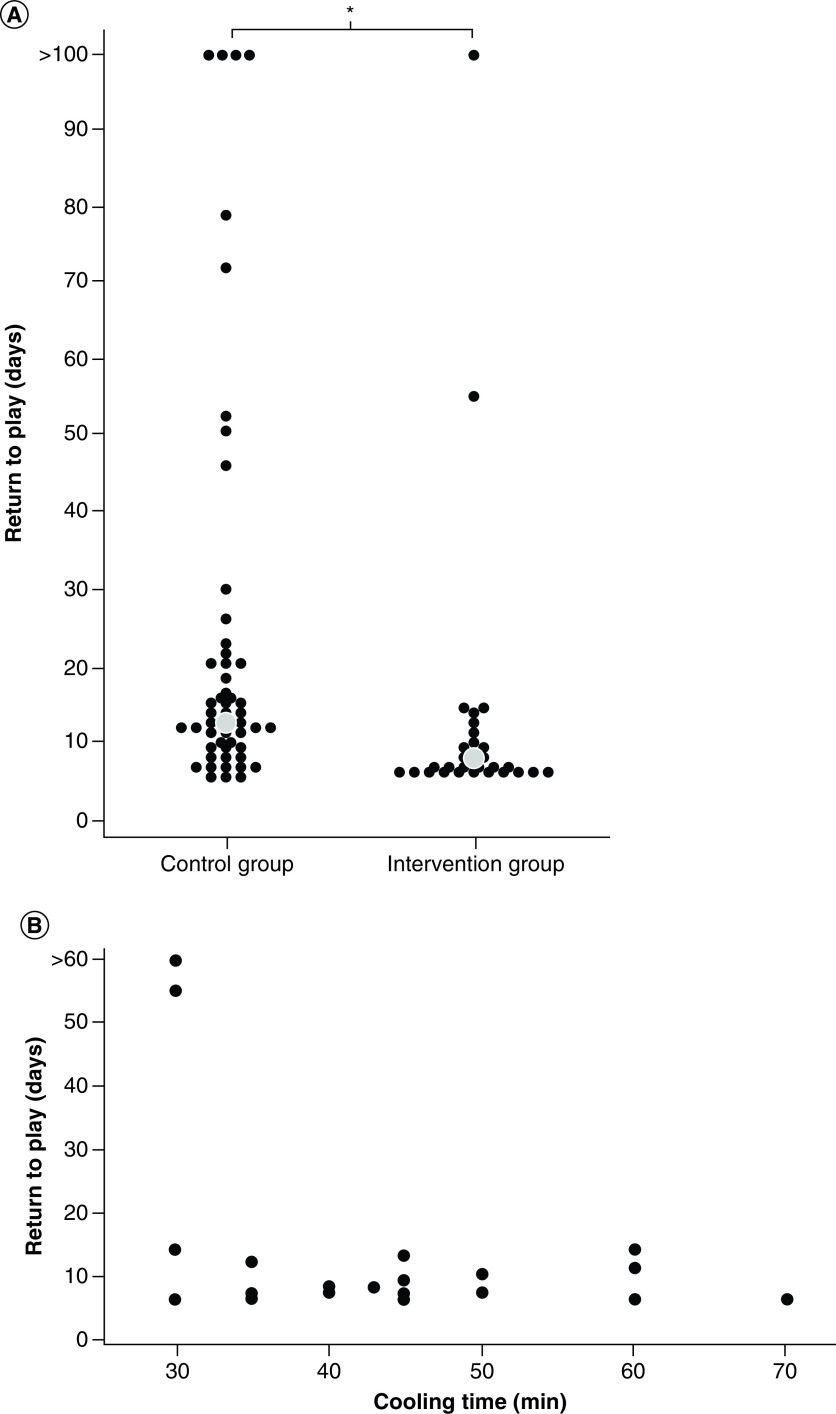
Return to play.

The median time to RTP was 7 days in intervention (cooling) group and 12.5 days in the control group (p < 0.0001; Cohen's D = 0.455). The range of RTP was 6 to >100 days in both groups. The proportion of players who were out of play for ≥3 weeks after concussion was 25% (13 of 52) in the control group and 7% (2 of 29) in the intervention group, p = 0.07. Individual and median values for RTP of each group are shown in Figure 5A.

Cooling time did not significantly correlate with RTP (Figure 5B). Although there were no significant adverse effects from treatment, four reported blurred vision, headache, fatigue and/or “pressure against the head” – all symptoms of concussion. All players reported they would consent to future cooling therapy if deemed necessary.

**Figure 6.**
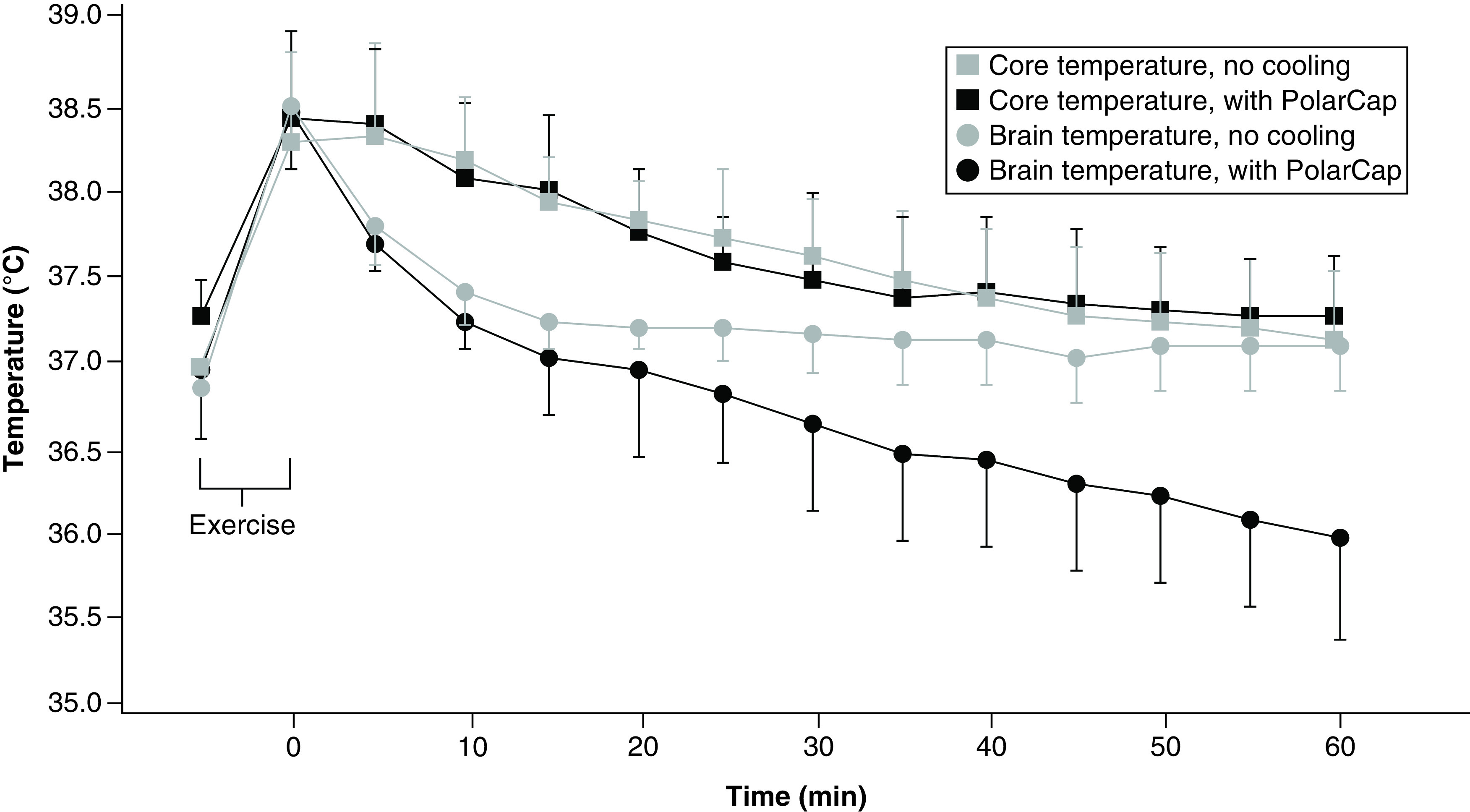
Core and subdermal forehead temperatures.

The subdermal forehead and core temperature of the volunteers reached 38.5–39.0°C after exercise in the pilot study. Immediately after exercise ended, subdermal forehead temperatures decreased in both groups. However, after initiation of selective head–neck cooling the subdermal forehead temperatures were 1.1°C lower than when the device was not (Figure 6).

## Discussion

The major finding is that ice hockey players attaining SRC and receiving selective head–neck cooling immediately postinjury had a significantly more rapid recovery to full game play than those receiving a standard SRC management protocol.

In the sports setting, systemic approaches (cooling blankets, gastric lavage, intravascular methods, ice baths etc.) are not feasible and may induce shivering, counteracting beneficial effects [41]. Other options, such as the insertion of a temperature-controlled balloon catheter into the nasal cavity [42], is not practical for use in a athlete who is conscious.

Instead, we aimed at selective head–neck cooling to rapidly reduce brain temperature. In previous studies, head–neck cooling lowered brain temperature without negatively influencing physiological and cognitive measures in healthy volunteers [43] and in patients with stroke or head injury [44]. A cooling cap used for 30 min was also found to reduce brain temperature with up to ~1–1.5°C [45]. When head–neck cooling was used for 30 min 5 ± 3 days post-SRC in 12 symptomatic 18- to 24-year-old college students, a partial but transient resolution of symptoms was observed. Another study of concussed athletes showed that a selective head–neck cap reduced symptoms [46].

In the present study, we used the PolarCap System and applied cooling of scalp and neck blood vessels in a procedure estimated to take ~30–60 min to reach desired temperature reduction, faster than the natural resolution of exercise-induced hyperthermia. Some athletes reported typical concussion symptom, hence no obvious side effects have been noted from the treatment, and it is not considered uncomfortable.

This small study has its limitations. There is currently much interest in improving SRC management in Sweden, based on the increased awareness of the problems associated with SRCs in ice hockey. It was assumed that players would not accept to receive standard care if another treatment with possible efficacy would be available and hence, we decided that treatment should be the same within teams. This study is not randomized, and we cannot fully exclude bias in terms of which team leader choose to participate – with or without the cooling – or not to participate. In view of the standardized treatment protocols and the educational strategies for SRCs conducted by the Swedish Ice Hockey League, each player was presumably subjected to similar initial management and RTP protocols. Moreover, we did not consider a ‘sham treatment’ (helmet worn without the cooling being turned on) feasible because it would be obvious to the participants whether the cooling was on or not. There was also a substantial variability in the number of SRCs between teams and seasons. In fact, two teams did not report a single concussion. Despite our best efforts, we cannot exclude some underreporting of SRCs by the medical teams. We used RTP as our main outcome measure. In several overviews, key factors predicting prolonged recovery post-SRC include female sex, a high number of previous concussions and marked initial symptom severity [47]. Here, the evaluation was performed in all-male teams. Furthermore, the mean number of previous concussions were identical between the groups. In fact, in contrast to the control group, there was no player in the intervention group who had never experienced a previous SRC. We did not have access to systematic reporting of the early SRC symptoms, for example, by the sports concussion assessment tool – 5 (SCAT5) [48], or biomarkers, and differences between the groups cannot be excluded. Furthermore, neuroimaging was not obtained. However, because the level of play was similar in both groups, marked differences in SRC severity are unlikely.

Plausibly, early cooling is warranted to attenuate the secondary injury cascades initiated immediately following SRC. The majority of players started cooling within 15 min postinjury, considered reasonable in view of the time required for player evaluation and treatment initiation. The duration of cooling has not been established, but based on previous work, a minimum of 30 min was selected [34]. In our pilot study of the three volunteers, subdermal forehead responded quickly to cooling. The sensor patch was placed in the forehead a few centimeters from the cooling system. Body and subdermal forehead temperatures decreased rapidly the first 30 min, and then slower until reaching ~37°C, supporting the rapid cooling. To avoid a direct effect of the cooling system on the sensors, we tried to isolate the sensors by placing them few centimeters away from the cooling cap, folded back the neoprene and constantly dried condensation away from the sensors throughout the experiment. Measurements with noninvasive ZHF thermometry (forehead temperature measurements by 3M Bair Hugger, MN, US) has provided good agreement with conventional core temperature measurements in many patient groups undergoing cardiac surgery [49,50], vascular surgery [50], gynecological and trauma surgery [51] and abdominal surgery [52] and during intensive care [53]. In previous reports [49,51], the mean temperature difference between the new ZHF sensor on the forehead and the pulmonary artery catheter and nasopharyngeal temperatures was found to be 0.23 and 0.07°C, respectively. Per reports from the manufacturer, the Bair Hugger ZHF sensor reaches a depth of 1–2 cm below the skin surface [49], thus reaching the superficial brain cortex. In a recent study, a simultaneous comparison between a ZHF sensor and a thermocouple inserted into the brain parenchyma of a pig model was made during selective brain cooling. There was a good agreement [40] between the two methods, showing an average of ∼0.5°C difference. To validate the ZHF thermometry readings, brain temperature needs to be measured invasively by implanting sensors into the brain parenchyma or with MRI. Using the current methods, such comparisons are neither possible nor ethical. Regardless, our results indicate that rapid, selective head–neck cooling may reduce time to reach normothermia after exercise.

## Conclusion

In the present pilot study, 81 professional ice hockey players sustained a sports-related concussion, of whom 29 received acute, selective head–neck cooling for >30 min, whereas 52 received standard management. In those receiving cooling intervention, a significantly shorter RTP was observed. Although some players reported symptoms commonly associated with SRC during cooling, no adverse effects attributed to the treatment *per se* were reported. In view of its safety and the evidence of the marked neuroprotective mechanisms in the experimental setting, selective head–neck cooling holds much promise for use in the early management of SRC to facilitate faster recovery. In this study, we show that selective head–neck cooling is a practical method, facilitating rapid normothermia after SRC and promoting a faster recovery. Larger studies are needed to define the role of selective head–neck cooling in acute SRC management and to determine the optimal cooling time needed to maximize benefit for the concussed player.

## Future perspective

Treatment options are limited in the acute management of sports-related concussions (SRC). A rapid decrease in brain temperature as early as possible after an SRC may attenuate secondary injury mechanisms and thus improve outcome. The present results suggest that rapidly induced brain normothermia using a device available at the sideline, shortened return-to-play after SRC in elite hockey players. These results should be confirmed in larger studies, preferably using biomarkers and/or neuroimaging.

**Summary points**

- Sports-related concussions (SRCs) are a significant health concern, particularly in ice hockey, and there is growing evidence that repetitive mild traumatic brain injury can cause long-term change in brain structure and function. Available treatment options are limited.
- Hypothermia with lowered brain temperature is neuroprotective and has been evaluated as a treatment modality in a variety of brain injuries.
- We addressed the hypotheses that immediate controlled head and neck cooling could hasten return-to-play in a Swedish cohort of concussed professional ice hockey players.
- The purpose of the clinical trial was to investigate how quickly the player travels through the obligatory graduated return-to-play protocol after an SRC. The graduated return-to-play protocol is a rehabilitation process with a gradually increased activity that lasts for at least 6 days, where the last step involves full participation in training or matches.
- Participating teams (15 teams from the elite ice hockey leagues for males in Sweden) were given the option to participate in the study group (receiving selective head–neck cooling after SRC) or the control group (standard SRC management). SRC diagnosis was established according to the concussion in the sports consensus statement.
- The selective head–neck cooling was initiated at a mean of 12.3 ± 9.2 min after the concussion in 29 players, and 52 SRC controls received standard management.
- The results from this study showed significant benefits of cooling in treating concussions with the head and neck cooling technology. The median time to return to play for the players who underwent cooling was 7 days and 12 days for those who did not. Two of 29 players (7%) who received head–neck cooling were out of play for ≥3 weeks, in contrast to 13 of 52 (25%) in the control group.
